# The response to unfolded protein is involved in osmotolerance of *Pichia pastoris*

**DOI:** 10.1186/1471-2164-11-207

**Published:** 2010-03-26

**Authors:** Martin Dragosits, Johannes Stadlmann, Alexandra Graf, Brigitte Gasser, Michael Maurer, Michael Sauer, David P Kreil, Friedrich Altmann, Diethard Mattanovich

**Affiliations:** 1 Department of Biotechnology, BOKU-University of Natural Resources and Applied Life Sciences, Vienna, Austria; 2 Department of Chemistry, BOKU-University of Natural Resources and Applied Life Sciences, Vienna, Austria; 3 School of Bioengineering, University of Applied Sciences FH-Campus Wien, Vienna, Austria; 4 Chair of Bioinformatics, BOKU-University of Natural Resources and Applied Life Sciences, Vienna, Austria

## Abstract

**Background:**

The effect of osmolarity on cellular physiology has been subject of investigation in many different species. High osmolarity is of importance for biotechnological production processes, where high cell densities and product titers are aspired. Several studies indicated that increased osmolarity of the growth medium can have a beneficial effect on recombinant protein production in different host organisms. Thus, the effect of osmolarity on the cellular physiology of *Pichia pastoris*, a prominent host for recombinant protein production, was studied in carbon limited chemostat cultures at different osmolarities. Transcriptome and proteome analyses were applied to assess differences upon growth at different osmolarities in both, a wild type strain and an antibody fragment expressing strain. While our main intention was to analyze the effect of different osmolarities on *P. pastoris *in general, this was complemented by studying it in context with recombinant protein production.

**Results:**

In contrast to the model yeast *Saccharomyces cerevisiae*, the main osmolyte in *P. pastoris *was arabitol rather than glycerol, demonstrating differences in osmotic stress response as well as energy metabolism. 2D Fluorescence Difference Gel electrophoresis and microarray analysis were applied and demonstrated that processes such as protein folding, ribosome biogenesis and cell wall organization were affected by increased osmolarity. These data indicated that upon increased osmolarity less adaptations on both the transcript and protein level occurred in a *P. pastoris *strain, secreting the Fab fragment, compared with the wild type strain. No transcriptional activation of the high osmolarity glycerol (HOG) pathway was observed at steady state conditions. Furthermore, no change of the specific productivity of recombinant Fab was observed at increased osmolarity.

**Conclusion:**

These data point out that the physiological response to increased osmolarity is different to S. *cerevisiae*. Increased osmolarity resulted in an unfolded protein response (UPR) like response in *P. pastoris *and lead to pre-conditioning of the recombinant Fab producing strain of *P. pastoris *to growth at high osmolarity. The current data demonstrate a strong similarity of environmental stress response mechanisms and recombinant protein related stresses. Therefore, these results might be used in future strain and bioprocess engineering of this biotechnologically relevant yeast.

## Background

The response of cells to high osmotic pressure and increased salinity has been a subject of close investigation in many different organisms [1-4]. Depending on the intensity of the osmotic shock the immediate response to high osmolarity usually includes the activation of the environmental stress response (ESR) and of the high osmolarity glycerol (HOG) pathway to induce changes that are necessary to cope with this stressful environmental condition in *Saccharomyces cerevisiae *[5,6]. In batch culture, osmotic shock usually implies a temporary growth arrest to adapt the cellular metabolism [7]. Major adjustments of gene transcription in *Saccharomyces cerevisiae *and other yeasts include the induction of glycerol-3-phosphate dehydrogenase *GPD1 *transcription [8], transcriptional repression of the plasma membrane glycerol efflux channel *FPS1 *[2], but also the adjustment of ribosome biogenesis and the translation and protein folding machinery [9]. Glycerol production, but also the production of other small organic molecules, is induced in different yeast species to compensate variations of osmotic conditions [10]. Polyols, such as glycerol, pertain to a class of small molecules known as compatible solutes, which, in contrast to inorganic ions, can be safely accumulated and degraded in the cell without impairing cellular function or having detrimental effects on protein and nucleic acid stability [11]. Furthermore, biomass yield is reduced upon exposure to high osmolarity because of higher maintenance energy in both, batch and chemostat cultures [7,12]. However, it is known that after the immediate shock response, transcript levels of many stress responsive genes return to near basal levels after cells have adapted to the new environmental conditions [6].

The effect of osmolarity on cellular physiology is not only of particular interest for the basic research community. As biotechnological production processes aim at high cell and product concentrations, cultivation media usually employ high concentrations of nutrient salts and carbon source resulting in high osmolarities. Additionally, there is some evidence that exposure to osmotic stress can have a beneficial effect on recombinant protein production in bacterial, yeast and mammalian host organisms [13-16]. Unfortunately, the positive effect of increased osmolarity on heterologous protein production is, at least in mammalian cells, often cell line specific [17] and e.g. in case of the yeast *Pichia pastoris *it remains anecdotal. The genome sequence of *P. pastoris *has been recently published [18,19] and with a publicly available sequence at hand thorough physiological investigations and characterization of this biotechnologically relevant organism becomes feasible.

In this context the effect of osmolarity on the physiology of *P. pastoris *was analyzed in both a non-expressing wild type (wt) strain and a recombinant protein secreting strain. The protein secretion strain expressed the antibody Fab fragment 3H6 [20,21] under the control of the constitutive glyceraldehyde-3-phosphate dehydrogenase (GAP) promoter. The effect of osmolarity was monitored in steady state by applying chemostat cultivation in both strains. Although chemostat cultivation differs from batch and fed batch systems, which are usually applied for large scale production of recombinant proteins, long term suboptimal growth conditions as they occur during batch and fed batch cultivation can also be applied in steady state chemostat conditions [22]. Furthermore, chemostat cultivation offers the advantage that growth rate related effects, which otherwise would interfere with high throughput protein and mRNA analytics, can be avoided [23].

To analyze the effect of increased osmolarity on host cell physiology, 2D Fluorescence Difference Gel Electrophoresis (2D-DIGE) and DNA microarray analyses were applied. These techniques have already been successfully applied to monitor the effect of environmental factors, such as temperature and osmolarity in yeasts [6,24-26]. Furthermore, HPLC analysis was applied to analyze to intracellular polyol and trehalose contents.

The obtained data indicated an unfolded protein response (UPR) like response upon growth at increased osmolarity in the non-expressing wt strain of *P. pastoris*. In the recombinant protein secreting strain, the UPR was obviously already induced due to protein overexpression. The observed overlap of the response to increased osmolarity and the response to recombinant protein production, lead to less adaptations/changes upon high osmolarity on both, the transcriptome and proteome scale, in the Fab secreting strain than in the non-expressing wt strain.

## Results

### General characteristics of cultures at different osmotic conditions

Chemostat cultivations of *P. pastoris *were performed at three different osmotic conditions, which were achieved by different concentrations of KCl in the growth medium. This resulted in supernatant osmolarities of approximately 140, 850 and 1350 mOsmol kg^-1^, which will be named low, medium and high osmolarity thereafter. Samples were taken at steady state, which means fully adapted cells were analyzed. The characteristics of the chemostat cultures did not dramatically change upon cultivation at different osmotic conditions (Table 1). Biomass yield decreased with increasing osmolarity in the wt strain and Fab 3H6 producing strain. However, the decrease in biomass yield was only statistically significant (*p *≤ 0.05) between cultivations at low and high osmolarity in both strains. The amount of total protein secreted into the culture supernatant did not change upon higher osmotic pressure, but was generally higher in the Fab 3H6 production strain. Osmolarity of the growth medium did not significantly influence specific productivity (q_P_) of the Fab 3H6. Generally, increased osmotic pressure poses a severe stress condition to cells [2]. Although the osmolarity was increased approximately 6-fold and ten-fold in the current study, no decrease of cell viability was observed. More than 97% of the cells in chemostat culture represented viable cells throughout all cultivations (Table 1). According to flow cytometry data, increased osmolarity resulted in a decrease of the mean cell size as indicated by a decrease of the mean forward scatter of the cells (Table 1).

**Table 1:** Characteristics of *P. pastoris *X-33 grown in carbon limited chemostat cultures at different osmolarities.

Clone	Osmolarity [mOs kg^-1^]	YDM [g L^-1^]	Total protein supernatant [mg L^-1^]	viability [%]	forward scatter	qP FabYDM^-1^h^-1^
wt	149 +/- 11,4	27.7 +/- 0.3	0.38 +/- 0.01	97.5 +/- 0.3	443 +/- 95	-
wt	865.7 +/- 3,2	27.3 +/- 0.2	0.36 +/- 0.02	98.8 +/- 0.2	258 +/- 15	-
wt	1351.3 +/- 2,0	26.0 +/- 0.3	0.38 +/- 0.00	98.1 +/- 0.2	297 +/- 4	-
Fab	135.2 +/- 3.3	27.8 +/- 0.2	0.47 +/- 0.04	97.2 +/- 0.4	355 +/- 60	0.039 +/- 0.004
Fab	857.3 +/- 8.5	26.8 +/- 0.4	0.44 +/- 0.05	97.9 +/- 0.6	225 +/- 30	0.042 +/- 0.002
Fab	1352 +/- 10,1	24.9 +/- 0.3	0.45 +/- 0.05	97.8 +/- 0.5	206 +/- 12	0.047 +/- 0.006

wt represents the non-expressing wild type strain. Fab represents the recombinant protein producing strain. Osmolarity values represent actual measured values of the culture supernatant. +/- represents the standard error of the mean. - not applicable.

### Production of compatible solutes and trehalose in *P. pastoris *upon growth at different osmolarities

In yeasts, glycerol is a very common solute but other polyols such as arabitol, mannitol and erythritol are also produced in some yeast species [10]. To analyze whether *P. pastoris *produces any of these substances, cell extracts were analyzed by HPLC.

**Figure 1:**
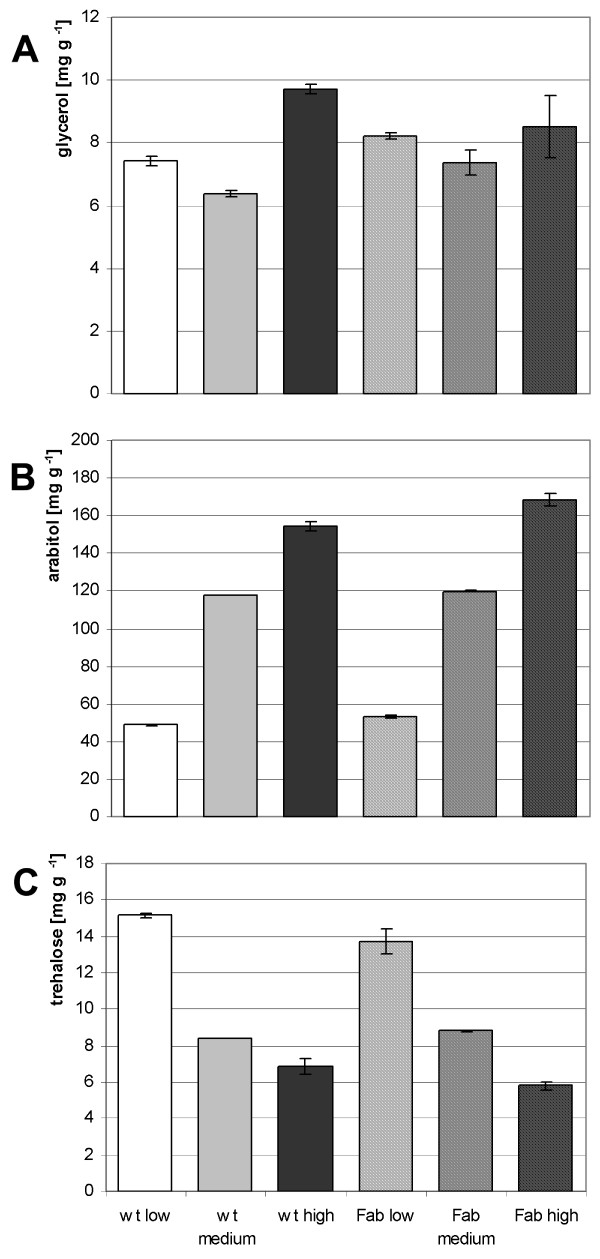
**Intracellular glycerol (A), arabitol (B) and trehalose (C) levels during carbon limited steady state cultivation of *P. pastoris *at different osmolarities (low, medium and high)**. wt - wild type strain. Fab 3H6 - Fab 3H6 strain. Error bars represent the standard error of the mean.

It turned out that very low levels of mannitol and nearly no detectable amounts of erythritol were present in *P. pastoris *cells (Additional file 1). Intracellular glycerol levels were higher than mannitol and erythritol levels and a slight significant increase in the wt strain from low to high and medium to high osmolarity occurred (*p *≤ 0.05), whereas no significant changes of glycerol content were observed in the Fab 3H6 expressing strain (Figure 1A). Surprisingly, arabitol was the most abundant compound of the analyzed polyols in *P. pastoris *cells (five fold higher basal level then glycerol) and showed statistically significant increased levels (*p *≤ 0.05) when shifting growth conditions towards high osmolarity (Figure 1B). A 3-fold increase of intracellular arabitol levels was observed when comparing low and medium osmolarity conditions and a 4-fold increase when comparing cells grown at low and high osmolarity.

Furthermore, intracellular levels of trehalose were analyzed as trehalose is thought to be involved in relieving or impeding protein folding stress [27], which may also occur during salt stress [9]. Intracellular trehalose levels were in the same range as glycerol levels but showed a significant trend (*p *≤ 0.05) towards decreased concentrations at medium and high osmolarity growth conditions in the wt strain but slightly missed the threshold *p*-value in the 3H6 Fab expressing strain (Figure 1C and Additional file 1)

### The effect of osmolarity on the *P. pastoris *intracellular proteome

As 2D-DIGE has already been successfully applied to monitor changes in the *P. pastoris *proteome upon growth at different temperatures [26], this method was also applied to track changes upon growth at elevated osmolarity. In the wt strain of *P. pastoris *approximately 300 proteins passed the criteria (see experimental procedures section for details), whereas about 150 proteins passed these criteria in the recombinant protein producing strain. Most of the protein spots represented low abundant proteins with too small quantities of proteins on the 2D gels to be confidently identified, resulting in 37 successfully identified proteins (Table 2). A list of all identified protein spots with corresponding peptides, obtained from MS/MS analysis, is available in Additional file 2. As already reported in previous studies [26,28], additionally to most likely full length proteins, protein fragments were identified according to the spot position on the gel (e.g Spot 4-Aco1, Spot 20 and 21-Ino1 and Spot 30-Ssb1; Table 2). Furthermore, protein levels showed the largest changes when low and medium and low and high osmolarity cultivations were compared, whereas only minor changes occurred when comparing medium and high osmolarity setpoints (see Additional file 3 for a complete table of relative protein levels).

**Table 2:** Proteins that were affected by growth at different osmolarities in carbon limited chemostat cultures of *P. pastoris *X-33.

				wt low/high		Fab low/high	
				
Spot no	Protein	description	MW/pI	Av ratio	1-ANOVA	Av ratio	1-ANOVA
1	Aco1	aconitase	84.5/5.93	-2.25	**3.70E-04**	1.01	**7.60E-04**
2	Aco1	aconitase	84.5/5.93	-1.13	5.80E-02	1.54	**4.40E-05**
3	Aco1	aconitase	84.5/5.93	-1.23	8.40E-02	1.49	**5.10E-04**
4	Aco1	aconitase	84.5/5.93	1.10	2.10E-01	1.51	**2.30E-04**
5	Agx1	alanine:glyoxylate aminotransferase	31.0/6.36	-1.79	**1.00E-04**	-1.43	**8.80E-03**
6	Aox1	alcohol oxidase	73.8/6.41	2.34	**1.90E-02**	-1.36	1.80E-01
7	Atp3	F1F0 ATPase subunit	31.6/7.74	3.28	**3.20E-06**	1.26	4.20E-01
8	Cdc19	pyruvate kinase	49.6/6.24	-1.81	**3.40E-03**	-1.26	**9.10E-03**
9	Cit1	citrate synthase	51.9/8.32	3.23	**2.90E-04**	-1.04	1.30E-01
10	Eft2	Elongation Factor 2	93.6/6.29	-1.93	**3.40E-04**	1.14	6.30E-02
11	Erg10	acetyl CoA acetyltransferase	41.7/6.10	-1.83	**7.00E-05**	-1.20	5.30E-02
12	Faa2	long chain fatty acyl-CoA synth.	25.4/6.73	-1.65	**3.40E-03**	1.16	5.10E-01
13	Fdh1	formate dehydrogenase	40.3/6.61	1.79	**1.30E-03**	1.35	**3.20E-03**
14	Gdh1	glutamate dehydrogenase	49.3/5.67	-2.38	**3.40E-02**	-1.45	**2.20E-04**
15	Gut1	glycerol kinase	68.2/5.33	1.31	**5.90E-03**	1.18	**1.20E-03**
16	Hbn1	nitroreductase (similar to bacterial)	21.8/6.30	1.12	2.10E-01	1.36	**3.00E-08**
17	Hsp60	heat shock protein 60	60.2/5.08	-1.83	**1.20E-05**	-1.13	**1.40E-04**
18	Icl1	isocitrate lyase	61.5/6.15	1.41	**2.10E-03**	1.56	**1.90E-05**
19	Ino1	inositol-1-P synthase	58.4/5.26	-2.61	**1.30E-05**	-1.88	**1.30E-04**
20	Ino1	inositol-1-P synthase	58.4/5.26	-1.78	**1.80E-05**	-1.11	**1.60E-04**
21	Ino1	inositol-1-P synthase	58.4/5.26	-1.06	3.60E-01	1.41	**9.00E-04**
22	Kar2	BiP	74.2/4.79	-2.95	**4.70E-05**	-1.05	5.90E-01
23	Pab1	poly A binding protein	68.6/5.07	-1.77	**5.00E-04**	-1.03	**3.70E-05**
24	Pdi1	protein disulfide isomerase	57.8/4.63	-1.66	**2.10E-05**	1.08	**1.00E-02**
25	Pgi1	phosphoglucose isomerase	61.9/5.83	1.12	1.00E-01	1.07	**7.00E-04**
26	Pgk1	phosphoglycerate kinase	44.1/7.77	-1.97	**3.30E-05**	-1.03	**2.40E-02**
27	Pil1	Primary component of eisosomes	35.3/5.03	1.35	**2.60E-02**	1.12	**6.20E-03**
28	Rib3	DHBP synthase/riboflavin	22.9/5.09	-1.84	**7.80E-06**	-1.16	7.60E-02
29	Sor2	similar to sorbitol dehydrogenase	38.6/5.76	1.34	**3.50E-02**	1.24	**2.50E-03**
30	Ssa4	heat shock protein	70.3/5.12	-1.20	**4.90E-02**	1.15	**1.10E-05**
31	Ssb1	heat shock protein	66.5/5.12	-1.02	8.70E-01	1.17	**1.70E-03**
32	Ssb1	heat shock protein	66.5/5.12	1.58	**1.20E-03**	1.03	**1.50E-05**
33	Ssc1	mitochondrial matrix ATPase	69.7/5.71	-4.7	**3.80E-03**	-1.20	**5.40E-05**
34	Sse1	hsp70 family ATPase	78.7/5.11	-2.79	**5.80E-05**	-1.14	**3.70E-04**
35	Ssz1	hsp70 family ATPase	57.9/4.83	-1.81	**8.00E-06**	-1.21	**4.30E-05**
36	Tfs1	carboxypeptidase Y inhibitor	24.2/4.92	-2.00	**3.90E-04**	-1.39	**2.00E-02**
37	Ymr090W	unknown function	25.1/6.91	-1.41	**3.40E-05**	-1.23	**1.30E-04**

Analysis was performed by 2D-DIGE and subsequent LC ESI-MS/MS identification. Protein standard name (according to the SGD, http://www.yeastgenome.org), protein functional description, theoretical molecular weight (MW) and theoretical isoelectric point (p*I*), average expression values and 1-ANOVA values are shown. Significant ANOVA values are indicated in bold letters. The average protein abundance fold change of the comparison of low and high osmolarity setpoints (Av ratio) are shown. Additional protein fold change data are available through Additional file 3.

**Figure 2:**
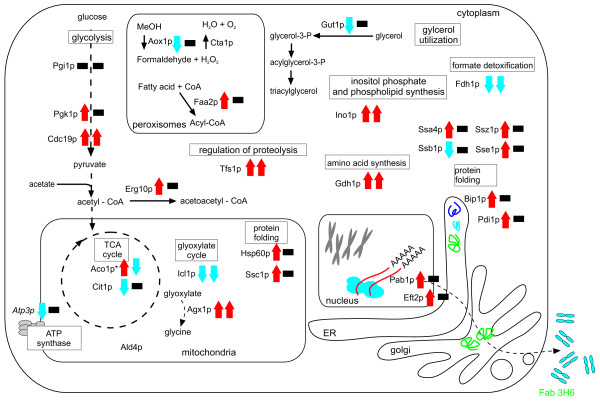
**Schematic representation of significant differences on the proteome level between high and low osmolarity cultivation of *P. pastoris *in carbon limited chemostats**. Left arrow represents changes in wt strain of *P. pastoris*, and the right arrow represents changes in the Fab 3H6 secreting strain. Red upward arrow - higher abundance at high osmolarity; Blue downward arrow - lower abundance at high osmolarity; black bar - no change in abundance.

As can be seen in Figure 2, the major impact of osmolarity on the *P. pastoris *proteome was on proteins involved in energy metabolism and protein folding. Whereas protein levels of a major spot of aconitate hydratase (Aco1p) were increased at medium and high osmolarity in the wt strain, they were not significantly affected by osmolarity in the Fab 3H6 producing strain. In contrast, in the Fab 3H6 expressing strain three minor isoforms or degradation products were significantly down-regulated at higher osmolarity, but showed no altered abundance in the wt strain. Furthermore, citrate synthase (Cit1p) protein levels were decreased at high salt concentrations in the wt strain but showed no significant change in the Fab producing strain. Formate dehydrogenase (Fdh1p), glycerol kinase (Gut1p), and isocitrate lyase (Icl1p) showed similar trends towards lower protein levels during medium and high osmolarity cultivations in both strains, whereas pyruvate kinase (Cdc19p) and phosphoglycerate kinase (Pgk1p) were generally up-regulated at higher osmolarity (it should be noted that Pgk1p levels returned to levels similar to low osmolarity cultivation in the production strain). Phosphoglucose isomerase (Pgi1p) showed lower levels at medium osmolarity in the production strain and a protein identified as Atp3p (a subunit of the mitochondrial F0F1 ATPase) was massively down-regulated at medium and high salt concentrations in the wt strain. Furthermore, alcohol oxidase (Aox1p), a key enzyme in methanol utilization, was down-regulated at medium and high osmolarity in the wt strain but did not show a significant change in the Fab 3H6 expressing strain. It should be pointed out that *AOX1 *transcription is thought to be repressed during growth on glucose and that the current study was performed with glucose as carbon source to constitutively express the heterologous protein under the control of the GAP-promoter. However, it was shown previously that basal levels of Aox1p were actually present during glucose limited growth of *P. pastoris *[26].

Similar discrepancies between the wt and the recombinant protein expressing strain were observed for proteins involved in protein folding and secretion and folding stress response. Whereas the major ER chaperone and unfolded protein response (UPR) sensor Kar2p/BiP and the protein disulfide isomerase Pdi1p were up-regulated at medium and high osmolarity in the wt strain, no changes of these two proteins were observed in the Fab producing strain. More prominently increased levels of cytosolic and mitochondrial chaperones Ssc1p, Sse1p, Ssz1p and Hsp60p were observed at medium and high osmolarity in the wt strain than in the Fab 3H6 producing strain. The stress induced chaperone Ssa4p showed increased levels at medium salt concentrations in both strains but returned to below basal levels at high salt conditions in the Fab producing strain. Ino1p, a protein involved in synthesis of inositol phosphates and inositol-containing phospholipids and which is linked to the UPR [29], was also up-regulated at high salt concentrations in both strains analyzed.

Other protein spots that changed their abundance upon cultivation at increased salt concentrations were Agx1p and Gdh1p (both involved in amino acid synthesis). Both of them showed higher protein levels during growth at high osmolarity.

Figure 2 summarizes the osmolarity-induced effects observed on the proteome level of *P. pastoris*.

### The effect of osmolarity on the *P. pastoris *transcriptome

To analyze the effect of different osmolarities on the *P. pastoris *transcriptome, *P. pastoris *specific microarrays were applied (Agilent platform). To support microarray analysis, real-time PCR was performed. Real-time PCR data proved to be consistent with microarray results (Additional file 4). More significant changes on the transcriptome level were observed in the wt strain than in the recombinant protein expressing strain (Table 3). Low *p*-values can result from a high technical variation within the replicates or reflect the biological truth within the samples. To determine if the lower amount of significantly regulated genes in the Fab expressing strain is a technical artefact, the correlation, standard deviation and the coefficient of variation for the replicates of the wt and expressing strain were compared. Correlation of intensity values was generally high between all microarrays of one group (wt/expressing red channel/green channel (see Additional file 5) with r^2 ^values between 0.95 and 0.97. The values for standard deviation and coefficient of variation (CV) indicated that the variance in replicates of the expressing strain was slightly but consistently higher than for the wt strain (on average CV 0.18 for the wt and CV 0.28 for the expressing strain, Additional file 5). Based on these results additional microarray experiments were performed to exclude any bias in the data. These additional data did not change the result or number of regulated genes, suggesting a true biological difference. To eliminate the possibility that the samples of the expressing strain vary more than the ones of the wt strain, hierarchical cluster analysis (HCA) and gene set analysis (GSA) were performed on the fold change data and indicated that regulation was indeed different in the two strains analyzed (Additional file 5).

**Table 3:** Number of regulated annotated genes (up- and down-regulated) in the wt strain and the Fab expressing strain at different osmolarities during carbon limited chemostat cultivation.

strain	low/high up	low/high down	low/medium up	low/medium down	medium/high up	medium/high down
**Wt**	226	165	153	50	1	1
**Fab**	27	13	13	10	8	6
**common genes**	22	7	11	4	0	0

Wt - non-expressing wild type strain. Fab - Fab 3H6 expressing strain. The number of genes that are similarly regulated in both strains are listed as common genes.

Because most of the genes that were regulated when comparing low to medium osmolarity were also regulated when comparing low to high osmolarity, the following data presentation and discussion will focus on the effects that were observed when low and high osmotic conditions were compared.

To get an overview of the general adaptations during steady-state cultivation, Fisher's exact test was performed to identify cellular processes, which were affected by different osmolarities on the transcript level. A total of 23 GO categories were either affected in both or at least in one of the analyzed strains (Additional file 5). Concordant with the mere number of regulated genes, there appeared more significantly affected cellular processes in the wt strain than in the heterologous protein expressing strain. Only 3 GO categories occurred to be affected in both strains, namely GO:0006811 (ion transport), GO:0007047 (cell wall organization) and GO:0019725 (cellular homeostasis). Additionally, in the wt strain the GO terms GO:0005975 (carbohydrate metabolism), GO:0006350 (transcription), GO:0006412 (translation) and GO:0042254 (ribosome biogenesis and assembly) were affected by increased extracellular osmolarity.

**Figure 3:**
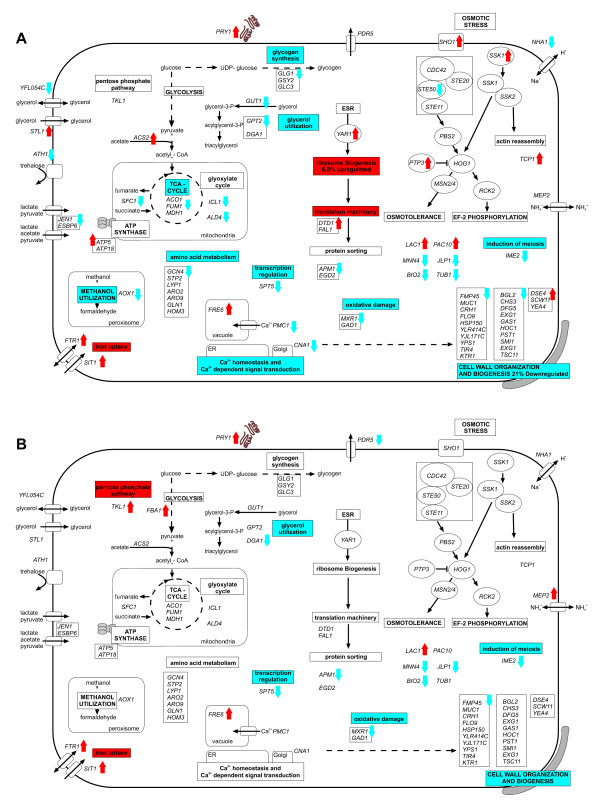
**Schematic representation of significant changes on the transcriptome level between high and low osmolarity cultivation of *P. pastoris *in carbon limited chemostats**. Wt strain (A). 3H6 Fab expressing strain (B). Only statistically significant genes are represented (cut-off *q *< 0.05). Blue downward arrows indicate down-regulation of genes at high osmolarity. Red upward arrows indicate up-regulated genes at high osmolarity. No arrow indicates no significant regulation of the genes or gene groups.

Figure 3 summarizes the important changes at the mRNA level of *P. pastoris*, grown in carbon-limited chemostat cultures when comparing high to low osmolarity, whereas microarray data for the discussed genes can be found in Additional file 6.

Regarding ion transport, uptake and metabolism, high osmolarity resulted in increased expression of the iron transporters *FTR1*, *SIT1 *and the vacuolar iron reductase *FRE6 *in both strains. Calcium ion homeostasis and calcium dependent signal transduction were obviously affected by high osmolarity in the wt strain as the Ca^2+ ^transporter *PMC1 *and Calcineurin A (*CNA1*) were down-regulated at high osmolarity.

**Figure 4:**
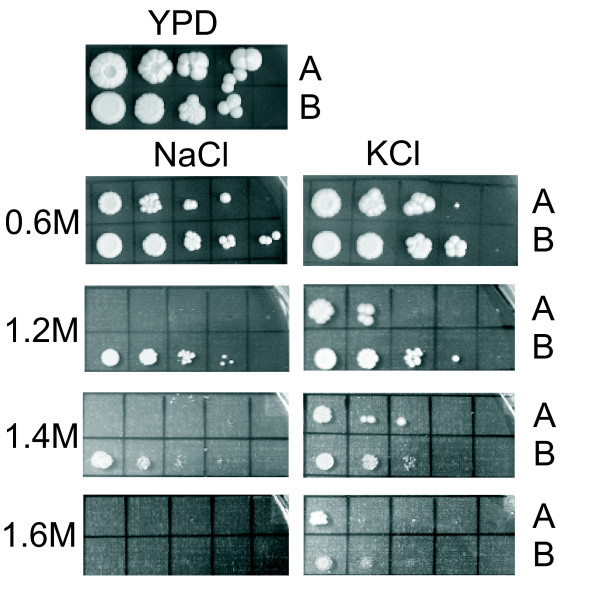
**Growth of *S. cerevisiae *HA232 (A) and *P. pastoris *X-33 (B) on YPD agar with different concentrations of NaCl or KCl**. YPD agar plates and plates containing 0.6 M NaCl or KCl were incubated at 28°C for 4 days, whereas all other plates were incubated at 28°C for 6 days.

A major effect was apparent for genes involved in cell wall organization and its biogenesis. Whereas 21% of the genes belonging to this GO group were down-regulated at high osmolarity in the wt strain, a similar effect, albeit with fewer significant genes, was observed in the 3H6 Fab secreting strain (Figure 4A and 4B). Additionally, a putative extracellular or cell wall associated protein with homology to the *S. cerevisiae PRY1 *gene was up-regulated in both strains at high osmolarity. Increased levels of a gene with homology to *S. cerevisiae PRY1 *upon increased salinity have also been reported for the halotolerant yeast *Hortaea werneckii *previously [30]. However, no changes in the protein pattern, indicating higher protein levels, were observed by SDS-PAGE of the culture supernatant (data not shown).

A signaling cascade for sensing and adaptation to osmotic stress in *S. cerevisiae *has already been established based on the available data [2,31] and genes with homology to the corresponding genes in *S. cerevisiae *were also identified in *P. pastoris*. None of the genes involved in osmotic stress sensing upstream of the mitogen activated protein kinase (MAPK) Hog1 showed significant regulation in the Fab 3H6 expressing strain, whereas *SHO1*, *SSK1 *and *PTP3 *were up-regulated and *STE50 *was down-regulated at increased osmolarity in the wt strain (Figure 4A).

Several genes involved in energy metabolism and storage carbohydrate metabolism were affected by increased osmolarity. *FBA1*, a key enzyme in glycolysis and gluconeogenesis, was up-regulated in the heterologous protein expressing strain at high osmolarity. The acetyl-coA synthetase *ACS2 *was up-regulated in the wt strain at high osmolarity. Transcript levels of several genes involved in the tricarboxylic acid (TCA) cycle and the glyoxylate cycle, namely *ACO1*, *FUM1*, *MDH1*, *SFC1 *and *ICL1 *were reduced and subunits of the ATP synthase (*ATP5 *and *ATP18*) were up-regulated during growth at high osmolarity in the *P. pastoris *wt strain. In the Fab producing strain, *TKL1*, involved in the pentose phosphate (PP) pathway was significantly up-regulated. Glycogen synthesis was also affected by high osmolarity as *GLG1*, *GSY2 *and *GLC3 *showed decreased transcript levels during growth at high osmolarity. Decreased levels of *DGA1*, *GUT1 *and *GTP2 *indicated changes in glycerol and lipid metabolism. Additionally, a homologue to the *S. cerevisiae *putative passive glycerol channel *YFL054C *was down-regulated and the active glycerol importer *STL1 *was up-regulated during growth at high osmotic conditions in the wt strain. Furthermore, significant down-regulation of the *P. pastoris *alcohol oxidase *AOX1 *was observed in the wt strain at high osmolarity, whereas no significant regulation was observed in the Fab 3H6 expressing strain. These data are concordant with data on the proteome level (Table 2).

In the wt strain approximately 7% of the genes involved in ribosome biogenesis and assembly were up-regulated during steady state cultivation at high osmolarity.

### The effect of recombinant protein production on the transcriptome of *P. pastoris *at low osmolarity

As the experiment was performed with a non-expressing wt and a recombinant protein expressing strain of *P. pastoris *the effect of recombinant protein expression itself on the cellular transcriptome at low osmolarity could also be analyzed. The most prominent effect of recombinant protein production on host cell physiology is the induction of the unfolded protein response (UPR) [32-35]. Therefore, we analyzed whether this effect could be observed at low osmolarity in glucose limited chemostat cultures of *P. pastoris*. It turned out that the transcriptional response to recombinant protein production during chemostat cultivation at low osmolarity was low. Only 7 mRNAs were significantly regulated when comparing the two strains using a strict cut-off (a Benjamini-Yekutieli corrected p-value of *q *≤ 0.05). Using a less strict cut-off (unadjusted *p*-value of ≤ 0.001) 79 genes were significantly regulated (Additional file 7). *HAC1 *was up-regulated in the Fab expressing strain and indicated the induction of the UPR in the Fab expressing strain. In contrast to *HAC1 *other members of the core UPR and targets of the *HAC1 *transcription factor showed no significant response by applying a *p*-value cut-off. However, Gene Set Analysis (GSA) applied on the microarray data revealed increased expression of genes related to the GO term GO:0006986 (Response to unfolded protein) (Additional file 7). The UPR (GO:0030968) is a specific response to unfolded protein in the ER and is a subcategory of GO:0006986.

The up-regulation of the *HAC1 *transcript as well as the significant upregulation of the response to unfolded protein indicated the induction of the UPR in the Fab 3H6 expressing strain. The effect was lower than in previous studies, which in fact were performed in non-carbon limited batch cultures. It is known that during carbon-limited chemostat cultivation of *S. cerevisiae *metabolic control can be more important than gene regulation [36]. Furthermore, it is known that part of the regulation of the UPR can be performed on a post-transcriptional or even post-translational level in *S. cerevisiae *and *Aspergillus niger *[37,38]. Less information about control mechanisms are available for *P. pastoris*. We conclude that similar to these previous studies, a substantial part of regulation, including the regulation of the UPR, is achieved on a post-transcriptional level during glucose-limited chemostat cultivation of *P. pastoris*.

Regarding the response to unfolded protein, GSA indicated significantly increased transcription of genes related to this GO category (GO:0006986) in the wt strain at high osmolarity. In contrast, higher growth medium osmolarity did not result in an induction of the response to unfolded protein in Fab 3H6 expressing strain (Additional file 5).

### Salt tolerance of Pichia pastoris

As no data on the salt tolerance of *P. pastoris *compared to *S. cerevisiae *were found in literature, a growth test on YPD agar plates, containing different amounts of NaCl or KCl, was performed. This growth test indicated higher tolerance to growth on NaCl and a less pronounced higher tolerance to growth on KCl of *P. pastoris *compared to *S. cerevisiae *(Figure 4).

## Discussion

### Production of Compatible Solutes

To counterbalance the osmotic pressure by high or low salt or solute concentrations in the growth medium, microorganisms produce various compatible solutes. In *S. cerevisiae *and many other organisms glycerol is the main osmolyte accumulated during osmotic stress. However, we found that intracellular glycerol levels were low at all osmotic conditions in *P. pastoris*. On the other hand, arabitol was more abundant than glycerol, even at low osmolarity, and it was accumulated in *P. pastoris *during growth at elevated osmotic pressure (Figure 1A and 1B). Glycerol production in *S. cerevisiae *depends on the increased expression of glycerol-3-phosphate dehydrogenase *GPD1 *and glycerol-3-phosphatase *GPP2 *[7,25]. Nevertheless, we could not find neither of the two genes involved in glycerol metabolism, *GPD1 *and *GPP2*, to be up-regulated on the transcript level and did not identify protein spots with altered expression which would match these two genes in *P. pastoris*. Furthermore, it was shown in *Debaryomyces hansenii *that NaCl stress lead to increased levels of proteins involved in the upper part of glycolysis and down-regulation of proteins involved in the TCA-cycle [24]. It was concluded that these changes may favor the accumulation of dihydroxyacetonephosphate and consequently the production of glycerol [39]. No changes related to the upper part of glycolysis were observed in the current study, although it is clear that the need for compatible solutes leads to a redirection of a part of the carbon source to alleviate the stress induced by increased osmolarity. This would make sense as arabitol obviously plays a more important role as compatible solute than glycerol in *P. pastoris*. However, the regulation of genes involved in glycerol uptake and efflux, such as the up-regulation of *STL1 *and the down-regulation of *YFL054C *may be beneficial for *P. pastoris *as well. The loss of minor osmolytes may result in detrimental effects on cellular integrity at high KCl concentrations. No significant regulation was observed for other putative glycerol transporters of *P. pastoris *recently described by Mattanovich and co-workers [18]. Arabitol synthesis is linked to the pentose phosphate (PP) pathway. However, no changes possibly linked to the PP pathway and arabitol synthesis were observed on the transcript or the proteome level. The regulation of arabitol synthesis might be mainly achieved on a post-transcriptional level by increased translation or by protein modification and changes of enzyme activity during chemostat cultivation of *P. pastoris*. Nevertheless, concordant with other studies, arabitol obviously is of particular importance for the metabolism of *P. pastoris *as it is also secreted into the supernatant at certain growth conditions, such as low oxygenation [40].

Trehalose has been previously shown to play an important role in heat shock induced refolding of proteins in baker's yeast [41] and *in vitro *[27]. Furthermore, trehalose may also be involved in the response to temperature induced stress in *P. pastoris *as intracellular levels increased at elevated temperature (own unpublished data). However, as trehalose levels were lower during growth at high osmolarity and no changes of the stress induced cytosolic trehalase *NTH1 *[42] were observed on the protein or mRNA level, trehalose may not be directly involved in the protection of proteins against osmotic induced protein denaturation or damage. It is more likely that, similar to *S. cerevisiae*, trehalose degradation may play a role during growth at elevated osmolarity [43], or that trehalose levels may be simply lower due to a redirection of carbon source to the production of arabitol rather then to the production of trehalose.

### Effect on Energy Metabolism

The differential response of Aco1p and the differences of transcript levels of genes involved the TCA cycle to different osmotic conditions between the wt and the Fab 3H6 expressing strain lead to the conclusion that recombinant protein production influenced the osmo-dependent adaptation of the energy metabolism. Previous data already indicated a metabolic burden and influence of recombinant protein production on energy metabolism in *P. pastoris *[26,44]. Furthermore, the key enzyme of methanol utilization, *AOX1*, was differently regulated in the two strains and indicated significant differences in the regulation of energy metabolism. Protein and transcript levels of the alcohol oxidase (*AOX1*) were significantly negatively affected by growth at high osmolarity in the wt strain but not in the Fab 3H6 secreting strain. *P. pastoris *Aox1 seems to be tightly regulated upon exposure to various stresses and might represent an ideal candidate as a marker gene/protein to monitor diverse external and internal stresses in *P. pastoris*. Apart from these additional data supporting the idea of a metabolic burden during recombinant protein production in *P. pastoris*, no clear interpretation about the changes of energy metabolism upon growth at different osmolarities in chemostat cultures emerged. Further investigations using a different approach to the one used in the current study will be necessary to elucidate the effect of osmolarity on the energy metabolism of *P. pastoris*.

### Activation of translation, ribosome biogenesis and the response to unfolded protein at high osmolarity

Another major effect was the massive increase of chaperones and UPR related proteins at high osmolarity. The UPR, including heat shock proteins and cellular chaperones, plays an essential role in the response to various stresses [45]. Apart from its role in the ESR of unicellular organisms, the UPR is also of great importance in human disease as highlighted by its involvement in the development of several human maladies such as diabetes, neurodegenerative disorders and cancer [46,47]. The observation of increased levels of molecular chaperones during growth at high osmolarity is concordant with previous results for *Aspergillus nidulans *[1] and similar to results obtained for *D. hansenii *[24] and *S. cerevisiae *[25] in batch culture. Furthermore, high osmotic pressure resulted in increased levels of Pdi1p and Kar2p, indicating Endoplasmic Reticulum related protein folding stress. Unlike *S. cerevisiae*, UPR induction has been reported to be a main event upon exposure to salt stress in the halotolerant yeast *Rhodotorula mucilaginosa *[9]. Generally, the induction of the UPR may not only be a result of high concentrations of ionic solutes such as salts but the response to unfolded proteins is also triggered by high osmotic pressure induced by other substances such as sugar compounds. It has been reported for mammalian cells that low as well as high hexose concentrations can lead to UPR induction [48,49]. The UPR has been described to be mainly a transcriptional response, but recently post-transcriptional and post-translational regulation has been described for fungal organisms [37,38]. In this context, mRNA levels of the UPR transcription factor *HAC1 *are increased in the Fab expressing strain as a reaction to recombinant protein production. On the other hand, GSA of the microarray data also showed induction of "responses to unfolded protein" at high osmolarity in the wt strain. These results are supported by the effects observed at the proteome level. A conventional induction of the UPR by increased *HAC1 *levels was not observed at increased osmolarity in neither of the strains. However, increased osmolarity resulted in increased Kar2p and Pdi1p on the proteome level in the wt strain. As this clearly demonstrated ER associated protein folding stress we refer to a UPR-like response of *P. pastoris *wild type cells at high osmolarity. Although a direct comparison between the wt and recombinant protein producing strain was not possible on the proteome level, this comparison was possible on the transcript level and strongly indicated the upregulation of processes involved in response to unfolded protein in the recombinant Fab producing strain. Thus, we hypothesize that the up-regulation of these ER resident proteins as well as other chaperones was obviously not necessary in a Fab 3H6 producing strain at high osmolarity, as these changes had already been triggered by the UPR that was induced by the recombinant protein.

Additionally to this UPR-like response, the induction of ribosome biogenesis and translation were apparent on the transcript level in the wt strain. The up-regulation of genes involved in protein synthesis during osmotic stress has been reported for the salt-tolerant yeasts *H. werneckii *and *D. hansenii *[30,50]. Furthermore, studies on brewing strains of *S. cerevisiae *concluded that the faster adaption to higher salt concentration compared to a laboratory strain was achieved by higher expression levels of genes involved in protein synthesis [51]. Similar to other environmental factors, such as temperature [52], translation might become a rate-limiting factor during growth at high osmolarities because of stress related to decreased intracellular water availability. Boosting the protein synthesis machinery might be necessary for growth of *P. pastoris *at elevated osmolarity. The Fab 3H6 expressing strain of *P. pastoris *did not show this increase of the protein synthesis machinery. We have shown previously that over-expression of the transcription factor *HAC1 *in *P. pastoris *batch cultures resulted in increased expression of genes involved in mRNA translation and to a massive increase of genes involved in ribosome biogenesis and assembly [53]. Obviously the up-regulation of ribosome biogenesis, translation and other co-regulated processes at high osmolarity was not necessary in the Fab 3H6 producing strain as these changes had already been induced by recombinant protein production itself.

This UPR-like response at high osmolarity also points to the fact that, similar to halotolerant yeast species like *R. mucilaginosa*, *P. pastoris *might use different mechanisms for gaining osmotic stress resistance than *S. cerevisiae*. This hypothesis was supported by the growth tests for salt tolerance, which were performed with *P. pastoris *and *S. cerevisiae*. *P. pastoris *showed indeed higher resistance to increased salt concentrations in the growth medium than *S. cerevisiae *(Figure 4). Many changes observed in the wt upon a change from low to high osmolarity were not observed in the recombinant protein expressing strain. Although high osmolarity triggered the response to unfolded protein, ribosome biogenesis and translation in the wt strain, the activation of these apparently co-regulated processes was compensated in the Fab 3H6 strain by recombinant protein induced UPR.

### Other cellular responses

Increased osmolarity also influenced other cellular mechanisms, such as some parts of the oxidative damage response, which apparently were not co-regulated with the protein synthesis and folding machinery. Therefore, these processes which seem to be an essential part of the response to increased osmolarity were monitored in both strains of *P. pastoris*. The interrelation of salt and oxidative stress is already established in plants [54] and the interrelation and cross-talk of the HOG pathway and other pathways such as protein kinase C (PKC) and calcineurin dependent signaling are also established in yeasts [2,55,56]. Changes in cell wall integrity signaling, which were evident by altered expression levels of cell wall components in both strains, may be directly related to the changes of the *CNA1 *and *PMC1 *transcripts, as some of these cell wall synthesis related genes are dependent on calcineurin signaling [56]. For example, decreased transcript levels of *CRH1 *and *GAS1 *at high osmolarity may indicate a change of cell wall rigidity at high osmolarity. High osmolarity results in decreased turgor pressure when compared with low or hypo-osmotic conditions. Thus, increased osmolarity results in cell shrinkage and smaller cells, which in fact was obvious by a decreased mean forward scatter of the cells in the present study (Table 1). In this context the down-regulation of cell wall components, which would result in lower cell wall rigidity, at high osmolarity makes sense. It has been shown in *Aspergillus nidulans *that salt addition to the growth medium resulted in decreased cell wall rigidity [57]. A further indication that high osmolarity is compatible with lower cell wall rigidity is the fact that the swollen cellular phenotype of Gas1 mutant cells, a gene which is also downregulated at high osmolarity in the current study, in *S. cerevisiae *is compensated by growth at high osmolarity [58]. Although this effect was very evident on the transcript level we were not able to monitor it on the proteome level. This may be simply due to the preparation of protein samples and the resulting absence of cell wall and membrane proteins, which are rather difficult to extract by standard protein preparation methods.

Additionally to these events, the induction of iron transporters at high osmolarity also occurred in both strains. A proteomic study of *Bacillus subtilis *recently highlighted that salt stress had an impact on iron homeostasis [59]. As already concluded for *B. subtilis*, also in *P. pastoris *the induction of iron uptake mechanisms might be of importance for growth in natural environments, where iron availability is generally scarce and may become even more limiting during growth implying high osmotic stress.

## Conclusion

Although the central ESR pathways are well conserved among fungi, the up- and downstream elements can be significantly different among species to satisfy niche-specific requirements [60]. Most notably, the presented data demonstrate a very high similarity and/or cross-talk of the stress induced by recombinant protein production and the reaction to elevated osmolarity in *P. pastoris*. Growth at high osmolarity resulted in the induction of the response to unfolded proteins. Additionally ribosome biogenesis and translation processes were upregulated, whereas genes involved in cell wall synthesis were downregulated at high osmolarity. Osmotic stress is a common condition for biotechnological production processes, due to high nutrient concentrations. In this light it is interesting to observe that *P. pastoris *is more osmo-tolerant than *S. cerevisiae*, and employs another main osmolyte, namely arabitol instead of glycerol, to compensate for osmotic stress.

The recombinant Fab 3H6 secreting *P. pastoris *strain was less prone to osmotic induced stress. Distinct differences, especially in the central carbon metabolism and processes linked to the UPR existed between the wt and the 3H6 Fab producing strain, which can be at least partially explained as response to unfolded protein is significantly induced in the Fab producing strain even at low osmolarity. Although in the current study elevated osmolarity did not result in increased productivity of recombinant Fab 3H6, the obtained data might be useful to explain the results of other research groups. It has been reported previously that osmotic stress applied prior to induction of protein secretion resulted in higher levels of scFv antibody in *P. pastoris *in batch culture [13]. Because osmotic stress obviously results in a UPR-like response in *P. pastoris*, it seems plausible that cells may be preconditioned for recombinant protein production as folding competence of the host cells may be increased compared to untreated cells. In this respect, the data obtained in the present study might be exploited not only for improved bioprocesses, but also for novel routes of strain engineering.

According to the data presented in this study, post-translational control mechanisms play an essential role in *P. pastoris*, especially during chemostat cultivation. Other proteomic methods such as the analysis of the phosphoproteome [61] might be very useful to gain detailed insight into these yet non-established mechanisms. However, the current data represent a first step towards a systems wide approach to assess the response to environmental stresses, as well as their overlap with recombinant protein induced stress, in *P. pastoris*.

## Methods

### Materials

All chemicals for yeast cultivations were molecular biology grade and were purchased from Roth, Germany. All chemical reagents for two-dimensional gel electrophoresis were high purity grade and were purchased from Sigma, unless stated otherwise.

### Yeast Strains

Two strains, which have been described recently [26], have been used in this study. For secreting the Fab 3H6, both the light and the heavy chain of the Fab fragment were expressed under the control of the constitutive GAP-promoter using the pGAPZαA vector. Secretion was mediated by the *S. cerevisiae *α-mating factor secretion signal. For the non-expressing strain, *P. pastoris *X-33 was transformed with an empty pGAPZαA vector as described by Gasser and co-workers [62].

### Chemostat cultivation

For chemostat cultivations a 3.5 L bench-top bioreactor (MBR, Switzerland) was used at a working volume of 1.5 L. A 1000 mL shake flask containing 150 mL YPG medium (2% (w/v) peptone, 1% (w/v) yeast extract, 1% (w/v) glycerol) was inoculated with 1 mL cryostock of the respective *P. pastoris *clones. The cultures were grown for approximately 24 h at 28°C and shaking at 170 rpm, before they were used to inoculate the bioreactor to an optical density (OD_600_) of 1.0. After a batch phase of approximately 24 hours the continuous culture was started at a dilution rate of D = 0.1 h^-1 ^(growth medium flow rate of 150 g h^-1^). pH was controlled at 5.0 with 25% ammonium hydroxide (w/w). Gas flow rate was kept constant at 1.5 vvm (volume gas per volume medium and minute) and dissolved oxygen was kept at 20% by controlling the stirrer speed. Three chemostat media, with different osmolarities, were used.

Batch medium contained per liter: 39.9 g glycerol, 1.8 g citric acid, 12.6 g (NH_4_)_2_HPO_4_, 0.022 g CaCl_2_·2H_2_O, 0.9 g KCl, 0.5 g MgSO_4_·7H_2_O, 2 mL Biotin (0.2 g L^-1^), 4.6 mL trace salts stock solution. The pH was set to 5.0 with 25% (w/w) HCl. Osmolarity of the growth medium was controlled by KCl concentration. Chemostat medium contained per liter: 50 g glucose ·1H_2_O, 0.9 g citric acid, 4.35 g (NH_4_)_2_HPO_4_, 0.01 g CaCl_2_·2H_2_O, 1.7 (low) or 29.9 (medium) or 48.5 (high) g KCl, 0.65 gMgSO_4 _7H_2_O, 1 mL Biotin (0.2 g L^-1^), and 1.6 mL trace salts stock solution. The pH was set to 5.0 with 25% (w/w) HCl. Trace salts stock solution contained per liter: 6.0 g CuSO_4_·5H_2_O, 0.08 g NaI, 3.0 g MnSO_4_·H_2_O, 0.2 g Na_2_MoO_4_·2H_2_O, 0.02 g H_3_BO_3_, 0.5 g CoCl_2_, 20.0 g ZnCl_2_, 5.0 g FeSO_4_·7H_2_O, and 5.0 mL H_2_SO_4 _(95-98% w/w).

Three chemostat cultivations were performed for each strain, whereas the osmolarity regime was different for each cultivation to avoid adaptive evolution effects and sample bias due to long term cultivation [63]. Samples were taken at steady state after 8 residence times after a switch of culture medium. Biomass was determined by drying duplicates of 10 mL chemostat culture to constant weight at 105°C in pre-weight beakers. Samples for 2D-DIGE and DNA microarray analysis were taken from the chemostat and immediately frozen at -80°C until use, whereat the samples for transcript analysis were fixed with 5% (v/v) phenol/ethanol prior to freezing. Viability of cells was determined immediately after samples were taken from the chemostat on a FACSCalibur flow cytometer (BD Biosciences) and a cell viability kit (BD Biosciences) as described previously [64].

### Determination of culture supernatant osmolarity

To determine the actual osmolarity of the supernatant, supernatant samples were analyzed on a Semi-Microosmometer K-7400 (Knaur).

### Analysis of intracellular polyols and trehalose

To quantify intracellular levels of glycerol, arabitol, mannitol, erythritol and trehalose, heat extraction was performed as described by Philips and co-workers [65]. Cell pellets were resuspended in 0.5 M TrisCl pH 7.5, heated to 95°C for 10 min and centrifuged for 10 min to remove cell debris. Supernatants were kept for analysis via HPLC. Isocratic conditions, using 4 mM H_2_SO_4 _as solvent and a flow rate of 0.6 mL min^-1 ^on a Aminex HPX-87H column (Biorad) at 40°C and a Biologic DuoFlow (Biorad) combined with a Smartline RI Detector 2300 (Knauer) were applied to separate and analyze substances (Additional file 1). Concentrations were determined by external standard solutions. Solute concentrations were correlated with biomass.

### 2D Fluorescence Difference in Gel Electrophoresis (2D-DIGE) and protein identification

2D-DIGE and protein identification were essentially performed as described previously [26]. After adequate sample preparation, cleaning, quantification and Cy-dye labeling, proteins were separated on IPG DryStrips pH 3-11NL (GE Healthcare) on an IPGphor for a total of 65 kVh. 2^nd ^dimension separation was performed by SDS polyacrylamide gel electrophoresis on 12% polyacrylamide gels. Fluorescence gel images were taken at a resolution of 100 μm on a Typhoon 9400 Fluorescence scanner. The DeCyder Software package v.5 (GE Healthcare) was used to analyze the obtained gel images. Significantly regulated protein spots (fold-change ≥ 1.5, 1-way ANOVA ≤ 0.05 in at least one comparison of cultivation conditions and present on at least 80% of the spot maps) were picked from Coomassie stained gels and after a tryptic digest subjected to reversed phase capillary chromatography (BioBasic C18, 5 μ, 100 × 0.18 mm, Thermo) and ESI-MS/MS on a quadrupole time-of-flight (Q-TOF) Ultima Global (Waters Micromass) mass spectrometer. Mass spectra were analyzed either by using the Protein Lynx Global Server 2.1 software (Waters) or X!Tandem http://www.thegpm.org/tandem/. Only proteins identified by at least 2 peptides were considered to represent confident hits, except for Pdi1, which was verified by Western blotting [26].

### DNA microarray analysis

DNA microarray analysis was performed using *P. pastoris *specific microarrays (Agilent) as described by Graf and co-workers [53]. RNA was extracted from ethanol/phenol fixed cell samples. Reverse transcription and synthesis of Cy3/5 labeled cRNA was done using the Low RNA Input Two-Color Amplification Kit (Agilent). cRNAs were purified via RNeasy Mini spin colums (Qiagen). Quality of total RNA and labeled cRNA was confirmed on an Agilent Bioanalyzer 2100 and the RNA Nano 6000 Assay Kit (Agilent). RNA concentrations were determined on a ND-1000 (Nanodrop). After hybridization at 65°C for 17 h, slides were scanned on an Agilent MicroArray scanner and raw data were extracted using Feature Extraction v.9.1 (Agilent). Normalization steps and statistical analysis of microarray data, including Hierarchical cluster analysis, Fisher's exact test and Gene Set Analysis (GSA), were done using the R software package http://www.r-project.org. For identifying differentially expressed genes, the False Discovery Rate was controlled strongly less than 5% (*q *< 0.05) using a Benjamini-Yekutieli correction for multiple testing. For Fisher's exact test and GSA, 63 gene ontology terms were considered. This list of terms was compiled based on the GOslim annotation of the *Saccharomyces *genome database http://www.yeastgenome.org, where some of the larger categories were resolved at a finer gene ontology level. A threshold of *p *≤ 0.05 was chosen to be appropriate to identify significantly regulated GO categories. Microarray data are available in the ArrayExpress database http://www.ebi.ac.uk/arrayexpress under the accession number E-MEXP-2433.

### Real-Time PCR

To support microarray data, Real-time PCR was performed. Total RNA was reverse-transcribed using a Superscript III cDNA synthesis kit (Invitrogen). Quantity of cDNA was determined on a ND-1000 (Nanodrop). Real time PCR was performed using the SensiMix Plus PCR premix (GenXpress) on a Rotorgene 6000 (Corbett Life Sciences). The following target genes were selected for Real-time PCR analysis: *ACT1*, *AOX1*, *DGA1*, *GLG1*, *SIT1*, *PDI1*, *HAC1*, *3H6 Fab HC *and *3H6 Fab LC *(Additional file 4). Data were analyzed via the Rotorgene Software package and Microsoft Excel. *ACT1 *was chosen as reference to determine relative mRNA levels of the other genes.

### Growth tests on different salt concentrations

*P. pastoris *X-33 and *S. cerevisiae *HA232 http://www.biotec.boku.ac.at/acbr.html were grown in YPD medium (2% (w/v) peptone, 1% (w/v) yeast extract, 2% (w/v) glucose) at 28°C on a shaker at 170 rpm over night. Cultures were diluted to an OD of 0.1 in sterile PBS and 1:10 serially diluted in sterile PBS. 3 μL were spotted onto YPD agar plates (2% (w/v) peptone, 1% (w/v) yeast extract, 1% (w/v) agar, 2% (w/v) glucose) containing 0, 0.6, 1.2, 1.4 and 1.6 M NaCl or KCl. Plates were incubated at 28°C for 4 to 6 days.

### 3H6 Fab quantification

To analyze the 3H6 Fab produced during chemostat cultivation, a sandwich ELISA was performed as described in previous studies [62].

## Authors' contributions

MD performed chemostat cultivation, microarray analysis, Real-time PCR, 2D-DIGE, HPLC analysis and growth tests. JS performed protein identifications by LC-ESI-MS/MS. AG performed the statistical evaluation of the microarray experiments. MM set up media recipes for cultivation and assisted in chemostat cultivations. BG, MS and DM contributed to the design of the study and data interpretation. DPK contributed to the design and advised on the analysis of the microarray experiments. FA supervised MS/MS analytics. MD, BG and DM drafted the manuscript. DM conceived of the study. All authors read and approved the final manuscript.

## Supplementary Material

Additional file 1 **Determination of intracellular polyol and trehalose content in *P. pastoris *upon growth at different osmolarities**. contains data on methodology of HPLC measurements, retention times of analytes and analyte concentrations with corresponding standard errors of the mean. Click here for file

Additional file 2 **Table of peptides of interesting proteins identified by 2D-DIGE and LC-ESI-MS/MS**. contains a list of all peptides assigned to the proteins identified by 2D-DIGE and LC-ESI-MS/MS and described in the manuscript (Table 2). Additionally, scores and scoring schemes are indicated. Click here for file

Additional file 3 **Fold-change and one-way ANOVA data for all contrasts of the 2D-DIGE experiment**. contains 2D-DIGE data of all comparisons (low to medium, low to high and medium to high) of both strains. Tables contain protein master numbers, short protein names, protein descriptions, fold-changes and corresponding one-way ANOVA values as described for Table 2. Click here for file

Additional file 4 **Real-time PCR results of *P. pastoris *grown at different osmolarities**. contains detailed data on real-time PCR. Primers sequences, PCR conditions as well as result diagrams are included. Click here for file

Additional file 5 **Quality of microarray experiments and statistical test for osmolarity experiments**. contains supplemental data on microarray analysis: Signal intensity plots, correlation of intensities, standard deviations and variations of the microarray experiment. Additionally, results of Hierarchical Cluster Analysis (HCA), Gene Set Analysis (GSA) and Fisher's exact test for the different osmolarities are included. Click here for file

Additional file 6 **Microarray results of interesting genes**. contains gene expression results of interesting genes, which are described and discussed in the manuscript. Click here for file

Additional file 7 **Differences between the non-expressing wt strain and the Fab 3H6 expressing strain of *P. pastoris *at low osmolarity at the transcript level**. contains microarray data on the gene expression differences between the two strains. Spreadsheet 1 contains microarray data for differentially regulated genes (*p*-value ≤ 0.001). Spreadsheet 2 contains results of gene set analysis (GSA). Click here for file
