# Supplementary material for: The response to unfolded protein is involved in osmotolerance of Pichia pastoris
Source: BMC Genomics. 2010 Mar 26;11:207. doi: 10.1186/1471-2164-11-207 (PMC2867824; doi:10.1186/1471-2164-11-207)
Supplement: Additional file 1 — Determination of intracellular polyol and trehalose content in P. pastoris upon growth at different osmolarities. contains data on methodology of HPLC measurements, retention times of analytes and analyte concentrations with corresponding standard errors of the mean. [file 1471-2164-11-207-S1.PDF]

# - Additional file 1 -

Supplemental Data on Intracellular Polyol and Trehalose Concentrations

Cell pellets were respuspended in 0.5 M TrisCl pH 7.5 and heated to 95°C for 10min. Samples were placed on ice and after they cooled down, cell debris were removed by centrifugation at 4°C. Trehalose, mannitol, arabitol, erythritol and glycerol concentrations were determined on an Aminex HPX-87H column with 4mM H_2SO_4 as solvent. Concentrations in the samples were determined via external standard solutions of the corresponding substances and correlated with biomass concentrations.

Retention times at 40°C and a flow rate of 0.6mL min^{-1}		
	trehalose	08.76min
	Mannitol	10.15min
	arabitol	10.99min
	erythritol	11.78min
	glycerol	13.49min

Intracellular sugar - alcohols and trehalose per Biomass [µg g^{-1}] for all measured substances. +/- represents the standard error of the mean.

	[µg /YDM]				
Strain & Conditions	***mannitol***	***arabitol***	***erythritol***	***glycerol***	***trehalose***
**wt low**	3.51 +/- 0.20	48.75 +/- 0.39	1.06 +/- 0.03	7.43 +/- 0.15	15.15 +/- 0.13
**wt medium**	3.27 +/- 0.09	117.86 +/- 0.22	0.40 +/- 0.05	6.37 +/- 0.11	8.40 +/- 0.04
**wt high**	4.65 +/- 0.25	154.51 +/- 2.63	0.00	9.71 +/- 0.13	6.85 +/- 0.42
**Fab low**	3.45 +/- 0.18	52.84 +/- 0.86	0.68 +/- 0.07	8.24 +/- 0.10	13.71 +/- 0.67
**Fab medium**	2.77 +/- 0.29	119.71 +/- 0.58	0.00	7.37 +/- 0.42	8.79 +/- 0.007
**Fab high**	4.82 +/- 1.54	168.32 +/- 3.17	0.00	8.52 +/- 0.99	5.81 +/- 0.20
