# Supplementary material for: The response to unfolded protein is involved in osmotolerance of Pichia pastoris
Source: BMC Genomics. 2010 Mar 26;11:207. doi: 10.1186/1471-2164-11-207 (PMC2867824; doi:10.1186/1471-2164-11-207)

# - Additional file 4 -

# Real Time PCR

### Primer sequences used for real time PCR

| target gene | oligo name | sequence (5'-3') |
|---|---|---|
| ***ACT1*** | ACT1 fw | CCTGAGGCTTTGTTCCACCCATCT |
| | ACT1 bw | GCAACATAGTAGTACCACCGGACATAACGA |
| ***SIT1*** | SIT1 fw | GTGATTTCTTGTACACTGCTTT |
| | SIT1 bw | AATCTTCTAATACGGACCAAA |
| ***DGA1*** | DGA1 fw | CATTGGGTATTTCTTCTGTTT |
| | DGA1 bw | TTTTTCAGGACAATATCAATAGT |
| ***GLG1*** | GLG1 fw | ATGGCAGTCTTGACAGTTTT |
| | GLG1 bw | TTCTTCGTCCACCTTTTCT |
| ***AOX1*** | AOX1 fw | TACACCACCGCTCTTTTG |
| | AOX1 bw | TCTCGTAAGTGCCCAACTT |
| ***PDI1*** | PDI1 fw | GGAAAGGCCCACGATGAAGTTGTC |
| | PDI1 bw | GCATCCTCATCATTGGCGTAAAGAGTAG |
| ***HAC1*** | HAC1 fw | GCGGCCCATGCTTCCAGAGAG |
| | HAC1 bw | CGGTACCACCTAAGGCTTCCAACC |
| ***3H6 Fab HC*** | 3H6 HC fw | CTA TTA CTG TGC AAG AAC GTC |
| | 3H6 HC bw | GAG GAG GGT GCC AGG GG |
| ***3H6 Fab LC*** | 3H6 LC fw | CTCCAATCGGGTAACTC |
| | 3H6 LC bw | GTGACTTCGCAGGCGTAGACTTTG |

Real time PCR was performed on a Rotorgene 6000 (Corbett Life Sciences) as described in the material and methods section using the following cycling conditions:

Hotstart: incubation at 95°C for 10min

| | temperature [°C] | time [sec] |
|---|---|---|
| Melting | 95 | 15 |
| Annealing | 60 | 20 |
| Elongation | 72 | 15 |

Cycles 45

Fluorescence signals were acquired during the annealing step at 60°C for each cycle.

## Results Real time PCR

Values relative to *ACT1* are shown. Error bars represent the standard error of the mean. Grey bars represent the wt strain mRNA levels and white bars represent the Fab 3H6 producing strain mRNA levels.

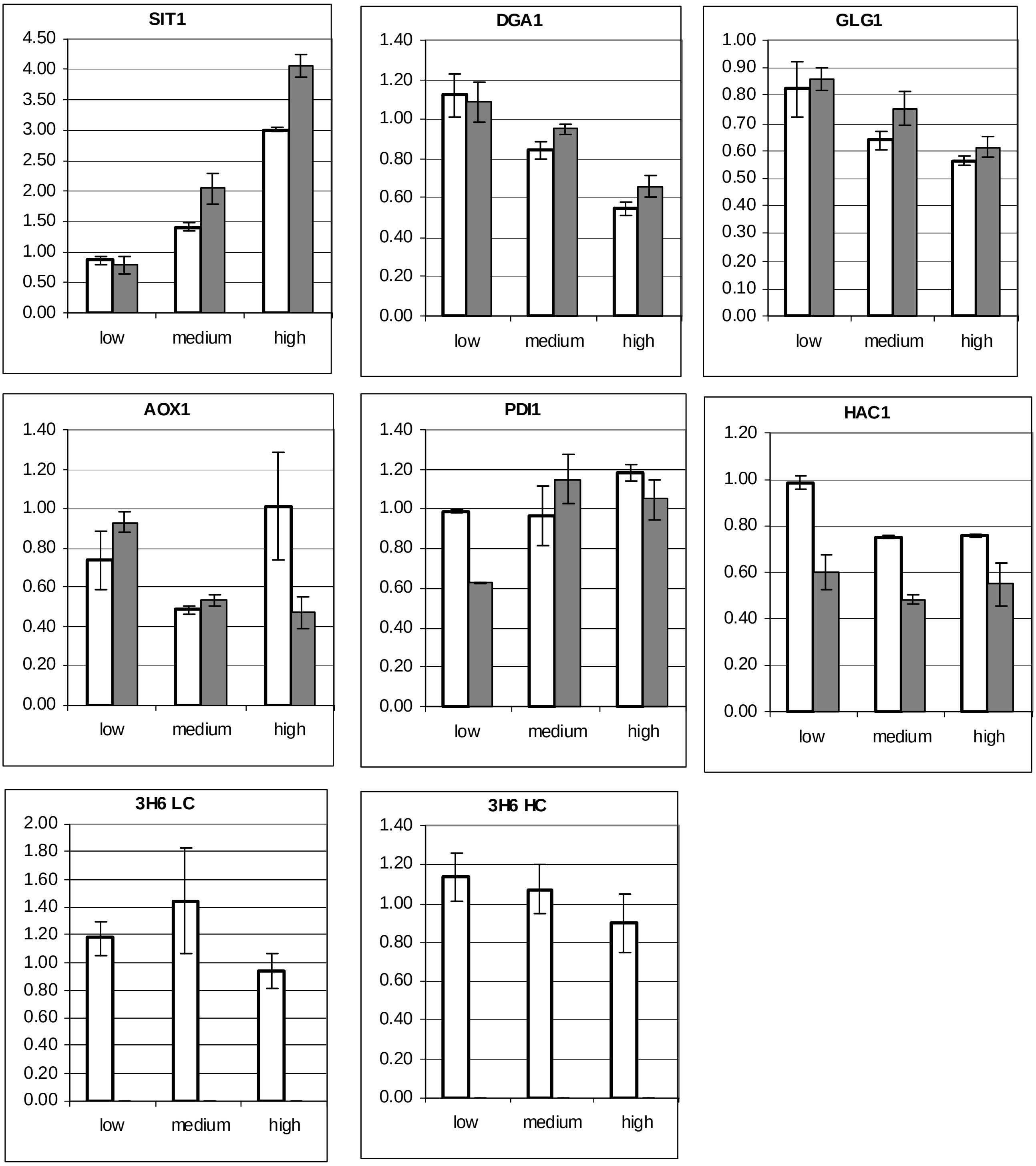

Supplement: Additional file 4 — Real-time PCR results of P. pastoris grown at different osmolarities. contains detailed data on real-time PCR. Primers sequences, PCR conditions as well as result diagrams are included. [file 1471-2164-11-207-S4.PDF]
