# Supplementary material for: The response to unfolded protein is involved in osmotolerance of Pichia pastoris
Source: BMC Genomics. 2010 Mar 26;11:207. doi: 10.1186/1471-2164-11-207 (PMC2867824; doi:10.1186/1471-2164-11-207)
Supplement: Additional file 5 — Quality of microarray experiments and statistical test for osmolarity experiments. contains supplemental data on microarray analysis: Signal intensity plots, correlation of intensities, standard deviations and variations of the microarray experiment. Additionally, results of Hierarchical Cluster Analysis (HCA), Gene Set Analysis (GSA) and Fisher's exact test for the different osmolarities are included. [file 1471-2164-11-207-S5.PDF]

Additional file 5

Microarray quality and statistical tests for different osmolarities

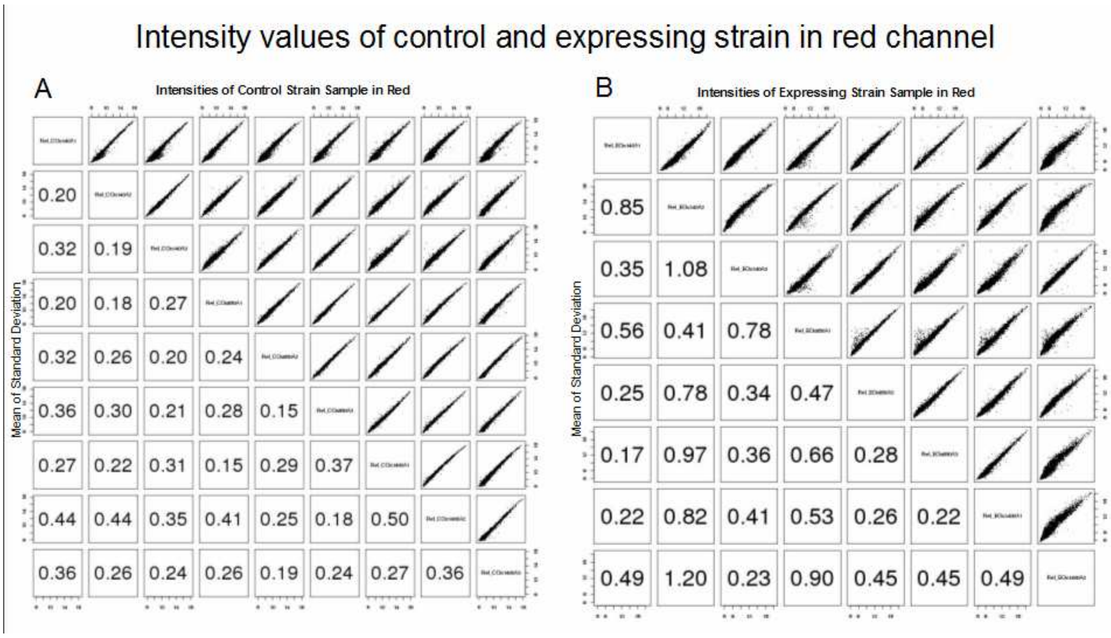

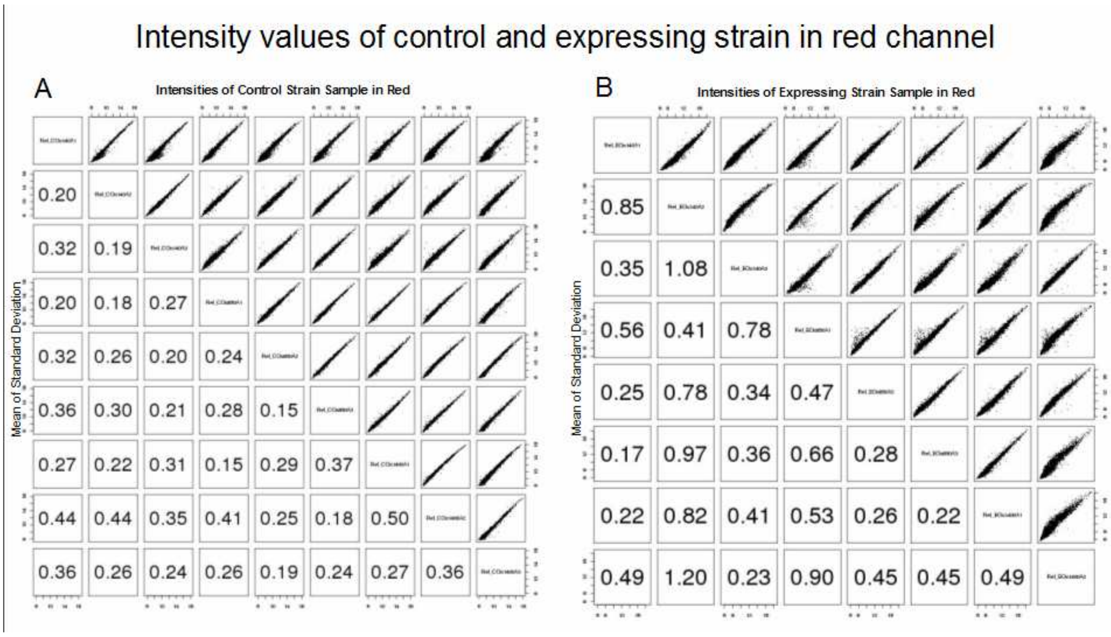

Signal intensity plots of the microarrays for the control and the expressing strain. Numbers represent the mean standard deviation for the individual comparisons.

Correlation of Intensities	r^2
Intensity wt (Red)	0.973002778
Intensity Fab (Red)	0.947978571
Intensity wt (Green)	0.972472222
Intensity Fab (Green)	0.972997222

Standard deviation within groups	Standard deviation
Intensity wt (Red)	0.28
Intensity Fab (Red)	0.54
Intensity wt (Green)	0.29
Intensity Fab (Green)	0.34
M wt (Red)	0.26
M Fab (Red)	0.65
M wt (Green)	0.39
M Fab (Green)	0.33

Variation within Replicates	Coefficient of variation
Intensities wt low	0.17
Intensities wt medium	0.19
Intensities wt high	0.17
Intensities Fab low	0.33
Intensities Fab medium	0.30
Intensities Fab high	0.22

The correlation, standard deviation and the coefficient of variation for the replicates of the wt and expressing strain.

Hierachical Cluster Analysis, Gene Set Analysis and Fisher's exact test

Hierachical clustering (HCA) of microarray results using the R statistical software package and pvclust. Distance: Euclidean

Cluster method: Ward

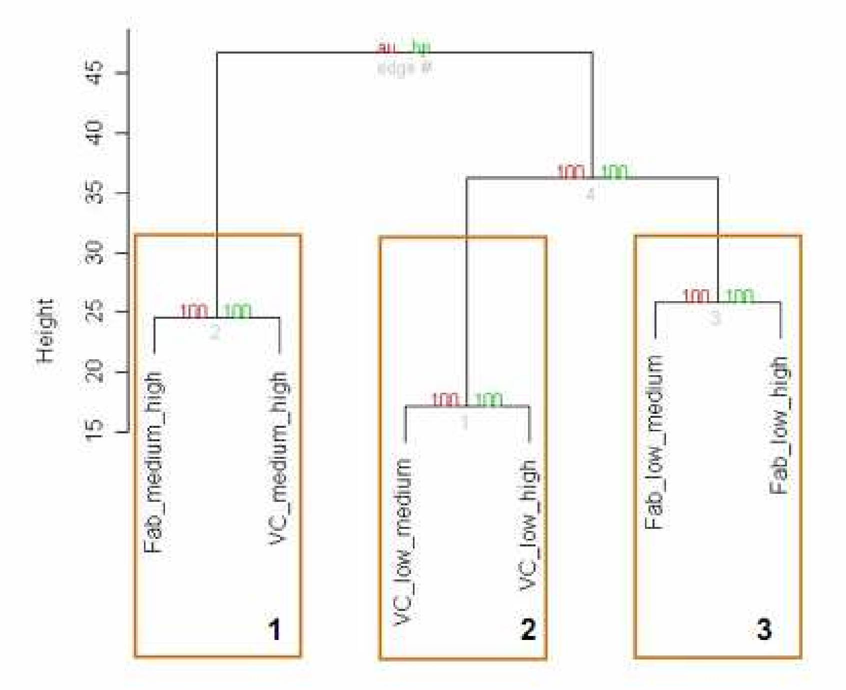

Dendrogram of the hierachical clustering result. VC – wt strain. Fab – Fab 3H6 expressing strain.

The comparison of medium and high osmolarity (where only minor transcriptional changes were observed) for both strains form a branch in the dendrogram. Furthermore, it can be seen that in the other two branches (2 and 3) that the two *P. pastoris* strains form separate branches in the dendrogram, indicating differential adjustments at the transcript level of the two strains when low/medium and low/high osmolarity cultivations were compared.

GSA shows that there is a significant difference between the control and the recombinant protein producing strain in many cellular processes such as carbohydrate and energy metabolism, the UPR but also ribosome biogenesis and assembly.

group	description	low<br>wt vs Fab	medium<br>wt vs Fab	high<br>wt vs Fab
GO:0005975	carbohydrate metabolic process		(-)0.01	(-)0.02
GO:0006091	generation of precursor metabolites and energy	(-)0.02		
GO:0006350	transcription			(+)0.04
GO:0006468	protein amino acid phosphorylation	(+)0.04		
GO:0006629	lipid metabolic process		(-)0.01	(-)0.05
GO:0006811	ion transport		(-)0.03	
GO:0006979	response to oxidative stress	(-)0.04	(-)0.03	(-)0.02
**GO:0006986**	**response to unfolded protein**	**(-)0.05**	**(-)0**	**(-)0.01**
GO:0007031	peroxisome organization and biogenesis			(-)0.04
GO:0007033	vacuole organization and biogenesis	(-)0.01		(-)0
GO:0007124	pseudohyphal growth		(-)0.02	
GO:0016044	membrane organization and biogenesis		(-)0.01	
GO:0016071	mRNA metabolic process	(-)0.02		
GO:0016568	chromatin modification	(+)0.04		(+)0.02
GO:0019725	cellular homeostasis	(-)0.01		(-)0.02
GO:0042221	response to chemical stimulus	(-)0.04	(-)0.02	
GO:0042254	ribosome biogenesis and assembly			(+)0.01

Gene set analysis (GSA) to identify differentially regulated GO groups between the wt strain (wt) and the Fab 3H6 (Fab) in carbon limited chemostat cultures of *P. pastoris* X-33 at different osmolarities. (-) down-regulated in control strain (+) upregulated in control strain. Numbers represent statistical significance by means of *p*-values.

		wt	wt	wt	Fab	Fab	Fab
**group**	**description**	**low/high**	**low/medium**	**medium/high**	**low/high**	**low/medium**	**medium/high**
GO:0000746	conjugation	(+)0.01		(+)0			
GO:0000910	cytokinesis					(-)0.04	
GO:0005975	carbohydrate metabolic process		(+)0.01			(-)0.03	
GO:0006399	tRNA metabolic process				(+)0.04	(+)0.01	
GO:0006457	protein folding			(-)0.04			
GO:0006468	protein amino acid phosphorylation			(+)0.01			
GO:0006519	amino acid and derivative metabolic process	(+)0.05	(+)0.01	(-)0.03		(+)0.05	(-)0.02
GO:0006629	lipid metabolic process						(+)0.05
GO:0006725	aromatic compound metabolic process				(+)0.05	(+)0	
GO:0006766	vitamin metabolic process				(-)0.03		(-)0
GO:0006811	ion transport				(-)0		(-)0.03
GO:0006970	response to osmotic stress				(-)0.05	(-)0.04	
GO:0006979	response to oxidative stress				(+)0	(+)0.05	(+)0.01
**GO:0006986**	**response to unfolded protein**	**(-)0.02**		**(-)0.04**			
GO:0007031	peroxisome organization and biogenesis				(-)0.03		(-)0
GO:0007033	vacuole organization and biogenesis						(-)0.01
GO:0007047	cell wall organization and biogenesis	(+)0.01	(+)0.02	(+)0	(+)0	(+)0.01	(+)0
GO:0007114	cell budding			(+)0.04			
GO:0007124	pseudohyphal growth			(+)0.02	(+)0	(-)0.02	(+)0
GO:0007126	meiosis			(+)0.02			
GO:0007165	signal transduction			(+)0.01			
GO:0009408	response to heat				(+)0.03		(+)0
GO:0015031	protein transport		(-)0.05	(+)0.03			
GO:0016072	rRNA metabolic process			(-)0.01			
GO:0016192	vesicle-mediated transport			(-)0.02			
GO:0016568	chromatin modification						(+)0.05
GO:0030435	sporulation			(+)0			
GO:0032196	transposition				(-)0.03		
GO:0042221	response to chemical stimulus	(+)0.02	(+)0.02	(+)0	(+)0	(+)0.01	(+)0
GO:0042254	ribosome biogenesis and assembly			(-)0			
GO:0042594	response to starvation	(-)0.04					
GO:0046483	heterocycle metabolic process					(+)0	
GO:0051169	nuclear transport					(+)0	
GO:0051186	cofactor metabolic process				(-)0.01		(-)0.01
GO:0051276	chromosome organization and biogenesis			(+)0.05			
GO:other	other				(+)0	(-)0.02	(+)0.05

Gene set analysis (GSA) to identify differentially regulated GO groups between different osmolarities in the wt strain (wt) and the Fab 3H6 expressing strain (Fab) in carbon limited chemostat cultures of *P. pastoris* X-33 at different osmolarities. (-) down-regulated in control strain (+) upregulated in control strain. Numbers represent statistical significance by means of *p*-values.

		wt LOW / HIGH		wt LOW / MEDIUM		wt MEDIUM / HIGH		FAB LOW / HIGH		FAB LOW / MEDIUM		FAB MEDIUM / HIGH	
**group**	**description**	**UP**	**DOWN**	**UP**	**DOWN**	**UP**	**DOWN**	**UP**	**DOWN**	**UP**	**DOWN**	**UP**	**DOWN**
GO:0000746	conjugation	**3.3E-02**	1.3E-01	**3.6E-02**	1.0E+00	1.0E+00	1.0E+00	1.0E+00	1.0E+00	1.0E+00	1.0E+00	1.0E+00	1.0E+00
GO:0005975	carbohydrate metabolic process	**2.9E-02**	7.0E-01	**8.3E-03**	6.4E-01	1.0E+00	1.0E+00	1.0E+00	5.7E-02	1.0E+00	1.0E+00	1.0E+00	1.0E+00
GO:0006259	DNA metabolic process	7.0E-01	**1.2E-02**	**4.6E-02**	1.0E+00	1.0E+00	1.0E+00	4.0E-01	1.0E+00	1.0E+00	1.0E+00	1.0E+00	1.0E+00
GO:0006350	transcription	**4.6E-02**	1.7E-01	6.7E-02	7.2E-01	1.0E+00	1.0E+00	6.3E-01	1.0E+00	1.0E+00	1.0E+00	1.0E+00	1.0E+00
GO:0006399	tRNA metabolic process	2.7E-01	1.0E+00	**8.6E-03**	1.0E+00	1.0E+00	1.0E+00	1.0E+00	1.0E+00	1.0E+00	1.0E+00	1.0E+00	1.0E+00
GO:0006412	translation	**1.3E-03**	**5.3E-05**	2.2E-01	3.5E-01	1.0E+00	1.0E+00	4.0E-01	1.0E+00	1.0E+00	1.0E+00	1.0E+00	1.0E+00
GO:0006468	protein amino acid phosphorylation	1.0E-01	**7.4E-04**	4.4E-01	6.4E-01	1.0E+00	1.0E+00	5.2E-01	1.0E+00	2.2E-01	1.0E+00	1.0E+00	1.0E+00
GO:0006519	amino acid and derivative metabolic process	**6.9E-04**	7.7E-01	**3.8E-07**	7.2E-01	1.0E+00	1.0E+00	1.0E+00	5.0E-01	1.0E+00	1.0E+00	1.0E+00	1.0E+00
GO:0006766	vitamin metabolic process	4.6E-01	1.0E+00	2.8E-01	1.0E+00	1.0E+00	1.0E+00	5.6E-02	1.9E-01	**6.8E-03**	1.0E+00	1.0E+00	1.0E+00
GO:0006811	ion transport	3.2E-01	2.0E-01	1.0E+00	**2.0E-02**	1.0E+00	**2.5E-02**	1.0E+00	**3.5E-03**	1.0E+00	**4.9E-02**	1.0E+00	1.0E+00
GO:0006970	response to osmotic stress	1.9E-01	1.0E+00	**4.0E-02**	1.0E+00	1.0E+00	1.0E+00	1.0E+00	1.0E+00	1.0E+00	1.0E+00	1.0E+00	1.0E+00
GO:0006974	response to DNA damage stimulus	1.0E+00	**3.4E-02**	6.3E-02	1.0E+00	1.0E+00	1.0E+00	1.0E+00	1.0E+00	1.0E+00	1.0E+00	1.0E+00	1.0E+00
GO:0006979	response to oxidative stress	2.7E-01	2.5E-01	4.3E-01	4.3E-01	1.0E+00	1.0E+00	**3.5E-03**	1.0E+00	1.0E-01	1.0E+00	1.0E+00	1.0E+00
GO:0007047	cell wall organization and biogenesis	**5.6E-07**	**3.0E-03**	**5.4E-05**	4.0E-01	1.0E+00	1.0E+00	**1.1E-03**	1.0E+00	1.0E+00	1.0E+00	1.9E-01	1.0E+00
GO:0007049	cell cycle	5.4E-01	**3.4E-03**	**8.2E-03**	3.6E-01	1.0E+00	1.0E+00	1.0E+00	1.0E+00	1.0E+00	1.0E+00	3.4E-01	1.0E+00
GO:0008150	biological process unknown	**2.9E-02**	9.2E-01	1.8E-01	9.3E-02	1.0E+00	1.0E+00	7.6E-01	2.0E-01	1.0E+00	1.0E+00	1.0E+00	1.0E+00
GO:0009653	anatomical structure morphogenesis	6.0E-01	**2.3E-02**	1.0E+00	4.0E-01	1.0E+00	1.0E+00	1.0E+00	1.0E+00	1.0E+00	1.0E+00	1.0E+00	1.0E+00
GO:0019725	cellular homeostasis	5.6E-01	2.9E-01	4.1E-01	9.9E-02	1.0E+00	**2.6E-02**	1.0E+00	**4.0E-03**	1.0E+00	5.1E-02	1.0E+00	1.0E+00
GO:0042221	response to chemical stimulus	**2.7E-02**	4.3E-01	1.2E-01	3.6E-01	1.0E+00	1.0E+00	7.7E-02	1.0E+00	4.3E-01	1.3E-01	3.5E-01	1.0E+00
GO:0042254	ribosome biogenesis and assembly	**5.3E-07**	**1.4E-05**	**8.6E-03**	1.0E+00	1.0E+00	1.0E+00	2.5E-01	6.2E-01	1.0E+00	1.0E+00	1.0E+00	1.0E+00
GO:0046483	heterocycle metabolic process	8.3E-01	1.1E-01	5.4E-01	6.1E-01	1.0E+00	1.0E+00	9.3E-02	1.0E+00	**1.2E-02**	1.0E+00	1.0E+00	1.0E+00
GO:0051186	cofactor metabolic process	6.1E-01	2.0E-01	1.0E+00	1.0E+00	1.0E+00	1.0E+00	1.9E-01	3.6E-01	**2.8E-02**	1.0E+00	1.0E+00	1.0E+00
GO:other	other	1.3E-01	6.3E-01	1.4E-01	1.3E-01	1.0E+00	1.0E+00	9.1E-02	4.5E-01	**4.7E-02**	1.0E+00	1.0E+00	1.0E+00

Gene ontology groups that appeared to be significantly regulated on the transcript level in at least on of the comparisons according to Fisher's exact test. wt – wild type strain. Fab – Fab 3H6 expressing strain. 'Low', 'medium' and 'high' represent the 3 osmolarity setpoints whereas 'up' and 'down' represent up- or down–regulated cellular processes. Significant p-values ($p \leq 0.05$) are highlighted in bold letters.
